# Development of Multifunctional Targeted Dual-Loaded Polymeric Nanoparticles for Triple-Negative Breast Cancer Treatment

**DOI:** 10.3390/pharmaceutics17040425

**Published:** 2025-03-27

**Authors:** Gantumur Battogtokh, Emmanuel O. Akala

**Affiliations:** Center for Drug Research and Development, Department of Pharmaceutical Sciences, College of Pharmacy, Howard University, Washington, DC 20059, USA; gantumur.battogtokh@howard.edu

**Keywords:** paclitaxel, cisplatin, dual-drug loading, copolymer, cetuximab nanoparticle conjugate, TNBC

## Abstract

**Background/Objectives**: Triple-negative breast cancer (TNBC) is a subtype of breast cancer that accounts for 15–20% of all breast cancer cases. TNBC is very difficult to treat with conventional treatment modalities such as chemotherapy, radiotherapy, and surgery; **Methods**: In this study, we developed a dual-loaded targeted nanotherapeutics against TNBC to solve the challenging problems associated with TNBC treatment: lack of efficacy, toxicity, and poor site-specific drug delivery; PEGylated methacrylate–polylactide copolymer containing cisplatin was synthesized and characterized; **Results**: The copolymer was used to fabricate nanoparticles (NPs) in the presence of paclitaxel with 1.33% drug loading. The nanoparticles were homogenous, with an average particle size of 198 nm and a negative zeta potential (−41.3 mV). Cetuximab (CTX), a monoclonal antibody that binds to the epidermal growth factor receptor (EGFR), was attached to the NP’s surface to enhance the targetability to TNBC. In vitro studies including cell uptake and cytotoxicity in MDA-MB-231 cells confirmed that CTX-targeted NPs have the potential for treating TNBC. The IC_50_ of CTX-NPs after 96 h of incubation was 0.1 μM, which was significantly lower than those of p-NPs (0.49 μM) and free drugs (PTX + cPt: 0.57 μM); **Conclusions**: In summary, this research shows that CTX-targeted polymeric NPs containing cisplatin and paclitaxel are effective in treating TNBC in vivo investigations are ongoing.

## 1. Introduction

Among various types of cancers, triple-negative breast cancer (TNBC) is known to be difficult to treat with conventional treatments, such as chemotherapy, radiotherapy, and surgery [1]. TNBC is a subtype of breast cancer and represents 15–20% of all breast cancers. It is triggered by many factors, including ethnicity, lifestyle, age, and genetics [2,3]. TNBC lacks receptors such as human epidermal growth factor receptor 2 (HER2), Estrogen receptor (ER), and Progesterone receptor (PR), which are expressed in breast cancers [4]. Although TNBC does not have the most common receptors expressed in breast cancers, it has some receptors, including EGFR (epidermal growth factor receptor) at 45–70%, PD-L1, CTLA-4, and PD-1 [5,6,7]. Additionally, drug resistance limits conventional therapies [5], and for this reason, monotherapy with a single agent has not been successful in the treatment of TNBC. Recently, many investigators have reported on combinations of bioactive agents against TNBC at both preclinical and clinical levels [1,8,9]. One of the advantages of combination therapy is that it allows cancer cells to be killed by different modes of action, resulting in improved therapeutic efficacy and avoiding overlapping toxicities [1]. Some of the combination therapies have reached clinical trials: chemotherapeutics [10,11,12], immunotherapeutic [13,14], PI3K/AKT/mTOR pathway inhibitors [15,16,17], and poly(ADP-ribose) polymerase (PARP) inhibitors [18,19].

The use of taxanes and platinum drugs is common for the treatment of TNBC, and these classes of chemotherapeutics are also very potent against other types of cancers [5]. The literature abounds in the combination of platinum and taxane drugs in the treatment of TNBC [20,21]. For example, Shi et al. demonstrated that cisplatin, paclitaxel, or nab-paclitaxel combinations could be the optimal first-line treatment of TNBC [22]. Previous reports demonstrated that some DNA-damaging agents, such as platinum drugs, may be more effective in TNBC tumors [23].

Various types of nanoparticles, such as polymer-based, inorganic-based, lipid-based, and protein-based, have been developed and applied in the field of drug delivery technology [24,25]. Among them, the lactide-based biodegradable polymeric nanoparticles that we are developing are more biocompatible and non-toxic compared to other nanoparticles, enhancing the chance of being approved quickly for clinical application by the FDA [26].

Several studies have been reported on the covalent conjugation of drugs to polymers and nanoparticles [27]. Conjugating drug molecules to a polymer backbone or nanoparticle prevents burst drug release, resulting in sustained drug release in a physiological environment. The drug-attached copolymer (containing a hydrophilic drug) can be used as a preformed polymer and a carrier for hydrophobic drugs in the fabrication of nanoparticles. Additionally, co-loading drugs into nanoparticles has advantages because the different modes of action of the drugs can solve the issue of drug resistance associated with a single drug. Here, we chose the DNA-damaging agent cisplatin and the microtubule-interfering agent paclitaxel as dual therapeutic agents to load in the nanoparticles.

The importance of multifunctional nanoparticles capable of site-specific drug delivery and overcoming drug resistance and side effects cannot be overemphasized. It is known that, at the moment, treatment of TNBC mainly relies on chemotherapy, which is associated with poor response, high toxicity, metastasis, relapse, and development of multidrug resistance [28,29,30]. Failure of chemotherapy, in addition to toxicity and resistance, may be due to the sequential mode of administration of all chemotherapy drug combinations. Thus, there is an urgent and unmet need for a novel targeted drug combination delivery system to achieve better outcomes for TNBC patients. The goal of this research is the development of multifunctional targeted nanoparticles capable of achieving better outcomes for TNBC patients using (a) targeted delivery of large doses of multiple drugs (cisplatin and paclitaxel) with different mechanisms of action into cancer cells to maximize therapeutic effects while reducing systemic toxicity (off-target toxicity); (b) EGFR-receptor-targeted nanoparticles containing a targeting ligand (cetuximab) that promotes binding and intracellular drug delivery by receptor-mediated endocytosis and that can bypass multidrug resistant protein (p-glycoprotein), which mediates the efflux of drug molecules; (c) the capability of long circulation without being sequestered into the liver [31]. Information in the literature describes the therapeutic application of cetuximab [32,33,34]; though we planned to use it as a targeting ligand, it could exert therapeutic effects.

Our strategy is to combine two commercially available anticancer agents (cisplatin and paclitaxel) into PEG–methacrylate–polylactide copolymer nanoparticles by introducing them via a covalent bonding (cisplatin) and hydrophobic interaction (paclitaxel), followed by antibody attachment to the surface of the nanoparticles.

## 2. Materials and Methods

### 2.1. Reagents and Materials

2-Hydroxyethyl methacrylate (HEMA) (97%), toluene (chromasolv, HPLC grade) (99.9%), calcium hydride, tin (II)-2 ethyl hexanoate (stannous octoate), chloroform-D, DMSO-D6 (dimethyl sulfoxide-d6), phosphorous pentoxide, anhydrous dichloromethane, Molecular sieves 4A° (1.6 mm diameter), Phenothiazine, Hydrochloric acid (ACS reagent, 37%), methanol (MeOH, ACS reagent, ≥99.8%), 1-(3-Dimethylaminopropyl)-3-ethylcarbodiimide hydrochloride (EDC-HCl), 4-Dimethylaminopyridine (DMAP), N,N-Dimethylformamide (DMF), tetrahydrofuran (THF), 4-Cyano-4-(phenylcarbonothioylthio)pentanoic acid (CPA), and 4,4′-Azobis(4-cyanovaleric acid) (ACVA) were obtained from MilliporeSigma (Burlington, MA, USA). Mal-PEG-MA (2 kDa) was obtained from Advanced Biochemicals (Lawrenceville, GA, USA). Chloroform (HPLC grade) was purchased from Fisher Scientific, Waltham, MA, USA, L(-)-Lactide was purchased from Polysciences, Inc. (Warrington, PA, USA). HEMA and toluene were distilled and analyzed for purity using ^1^H NMR. Cisplatin and paclitaxel were purchased from Adooq Bioscience (Irvine, CA, USA). Cetuximab was purchased from MedChemExpress (Junction, NJ, USA). The molecular weight of all synthesized polymers and macromonomers was analyzed by gel permeation chromatography (GPC) in the Reference Standard Laboratory (RSL) at USP (Rockville, MD, USA).

### 2.2. Oxidation of Cisplatin (cPt)

The oxidation of cisplatin was performed according to previous reports [35,36]. In brief, cisplatin (0.4 g, 1.32 mmol) was weighed into a round-bottom flask (50 mL), and 10 mL of deionized water (DIW) was added, followed by stirring at room temperature (RT) to make a suspension. To the suspension, 28 mL (20 times the excess) of hydrogen peroxide (30% *w v*^−1^, 24 mmol) was added and stirred. The reaction was carried out at 60 °C for 2 h to form a clear yellow solution. After completing the reaction, the liquid was collected into a 50 mL centrifugation tube and kept in the fridge overnight to recrystallize the resulting product. The precipitate was collected by centrifugation at 3500 rpm for 5 min. The residue was washed with cold water (40 mL), ethanol (40 mL), and diethyl ether (40 mL). After washing, the solid was dried in a vacuum oven overnight. The yield was 75%. The dried sample was examined using an FT-IR spectrometer.

### 2.3. Synthesis of Oxidized Cisplatin-Succinate Conjugate

The synthesis was accomplished using the methods previously reported with minor modifications [37]. Oxidized cisplatin (c,c,t-[Pt(NH_3_)_2_Cl_2_(OH)_2_], 0.3 g, 0.9 mmol) was weighed in a 50 mL round-bottom flask and dissolved in DMSO (16 mL) by stirring for 10 min at RT. The mixture was reacted with succinic anhydride (0.09 g, 0.9 mmol) by stirring at RT. After 24 h of reaction, the DMSO solution was mixed with 60 mL of deionized water (DIW; 5 times diluted). The mixture was kept at −70 °C overnight to freeze. The frozen sample was lyophilized for 2–3 days to remove the solvent. The yield was 80.1%. Confirmation of the reaction product was achieved through proton NMR, FT-IR, and ESI-MS spectrometer analysis. ^1^H NMR (400 MHz, δ, ppm, DMSO-d6): 2.38–2.41 (4H, m, CH_2_-CH_2_- of succinate), 5.81–6.07 (6H, m, NH_3_- of cisplatin). ESI-MS (1:1 = THF:MeOH with 10% of NaCl): calculated m/e 433 g mol^−1^; observed me^−1^ 456.96 g mol^−1^ [M + Na^+^].

### 2.4. Synthesis of HEMA-Poly (L(-)-Lactide Macromonomer (HEMA-PLA)

Recrystallized L(-)-lactide (12.0468 g, 0.0418 mol), HEMA (1.8 mL, 0.00738 mol), and stannous octoate (4–6 drops) were added to the round-bottom flask containing a magnetic stirrer. The round-bottom flask was kept under vacuum for 10 min, and polymerization was allowed in an inert atmosphere by continuously flushing with nitrogen gas for 24 h on a silicone oil bath maintained at 120 °C. After obtaining the white viscous product (polymer), it was dissolved in chloroform and extracted with 0.1 M HCl (3 times) to remove the catalyst and was rinsed with DIW. The pure polymer in the chloroform layer was precipitated with excess cold methanol, collected by filtration, and dried in a vacuum oven over phosphorous pentoxide. The reaction product HEMA-(poly (L(-)-Lactide macromonomer (HEMA-PLA) was analyzed by proton NMR, FT-IR, and GPC. ^1^H NMR (400 MHz, CDCl_3_, δ, ppm): 1.56–1.58 (d, CH_3_, lactide), 1.93 (s, 3H, methacrylate), 4.348 (m, 1H, CH, terminal lactide), 4.362 (m, 4H, CH_2_CH_2_, methacrylate), 5.130–5.182 (m, 1H, lactide), 5.59 (s, 1H, methacrylate), 6.111 (s, 1H, methacrylate). The molecular weight and polydispersity index of the macromonomer were determined by GPC (a Waters instrument equipped with a Refractive Index detector). For the determination of molecular weight, tetrahydrofuran (THF) was used as the mobile phase at a flow rate of 1 mL min^−1^ and at a temperature of 35 °C. Macromonomers were dissolved in THF, filtered, and then injected into two Agilent columns of Plgel MIXED with 5 μm, 300 × 7.5 mm. The average molecular weights were estimated by employing a series of polystyrene standards.

### 2.5. Synthesis of HEMA-PLA Macromonomer-Cisplatin Conjugate (HPLA-cPt)

The synthesis was accomplished using the methods previously reported with minor modifications [38]. cPt-succinate (200 mg, 0.435 mmol), EDC-HCl (0.868 mmol), and DMAP (0.435 mmol) were dissolved in 30 mL of round-bottom flask containing 2 mL of DMF (anhydrous) and stirred for 10 min. PLLA-HEMA (1 g, 0.435 mmol) was added to 1 mL of DMF and sealed and stirred under nitrogen for 20 min at RT, followed by stirring for 24 h at RT. The reaction mixture was poured into cold diethyl ether (DE, 200 mL) to precipitate, filtered using filter paper, and washed with DE (100 mL). The residue was dried in a vacuum oven over phosphorous pentoxide for 2 days. The yield was 90.2%. The reaction product was analyzed using proton NMR and FT-IR spectrometers. 1H NMR (400 MHz, δ, ppm, DMSO-d6): 1.476 (m, CH_3_, lactide), 1.87 (s, CH_3_, methacrylate), 2.98–3.03 (m, 4H, CH_2_CH_2_, succinate), 4.307 (m, 4H, CH_2_CH_2_, methacrylate), 5.216 (m, 1H, CH, lactide), 5.502 (s, 1H, methacrylate), 6.027 (s, 1H, methacrylate), 6.505–6.539 (6H, m, NH_3_- of cisplatin). The FT-IR spectrum shows significant functional groups: -NH, HO- (cPt-SA), and HO (PHPLA) at 3000–3500 cm^−1^, C-H at 2997.54 cm^−1^, C=O at 1779.03 cm^−1^, N-Pt at 1700 cm^−1^, C=C bond at 1648 cm^−1^, and -C-O-C at 1095 cm^−1^. The conjugate in THF was analyzed by GPC as described above.

### 2.6. Synthesis of Poly (Mal-Peg-HPLA-cPt) by RAFT (Reversible Addition Fragmentation Chain Transfer) Polymerization

The RAFT polymerization of methacrylate macromonomers was completed based on previous literature with modifications [37,39,40]. HPLA-cPt conjugate (500 mg, 0.181 mmol), mal-PEG-MA (36.2 mg, 0.0181 mmol), CPA (4-Cyano-4-(phenylcarbonothioylthio)pentanoic acid), and AVCA (4,4′-Azobis(4-cyanovaleric acid)) (1.267 mg, 0.0045 mmol) were weighed in a 30 mL round-bottom flask and dissolved in 3 mL of EtOH and 1 mL of DMF. The ratio of reagents was 10:1:0.5:0.25. The reaction mixture was stirred by a magnetic stirrer under nitrogen for 10–20 min and continued overnight on an oil bath maintained at 70–85 °C. After the reaction was completed, the mixture was dropped into cold diethyl ether (DE, 120 mL) to obtain poly (mal-Peg-HPLA-cPt) as a precipitate. The residue was collected on filter paper using vacuum filtration and washed with cold DE (100 mL), followed by drying in a vacuum oven in the presence of phosphorus pentoxide for 2–3 days. The yield was 78.1%. The poly-mal-Peg-HPLA-cPt (PHPLA-cPt) was characterized by proton NMR and GPC. ^1^H NMR (400 MHz, δ, ppm, DMSO-d6): 0.998 (m, CH_3_, methacrylate), 1.472–1.49 (m, CH_3_, lactide), 3.0–3.05 (m, 4H, CH_2_CH_2_, succinate-cPt), 3.521 (m, 4H, -O-CH_2_CH_2_-, PEG), 4.22–4.35 (m, 4H, CH_2_CH_2_, methacrylate), 5.212–5.23 (m, 1H, CH, lactide), 6.5–6.579 (6H, m, NH_3_- of cisplatin). The polymer in THF/toluene (1:1) was analyzed by GPC as described above. The FT-IR spectrum shows significant functional groups: -NH, HO- (cPt-SA), and HO (PHPLA) at 3000–3500 cm^−1^, C-H at 2997.54 cm^−1^, C=O at 1757.03 cm^−1^, N-Pt at 1700 cm^−1^, C=C bond at 1648 cm^−1^, and -C-O-C at 1089 cm^−1^.

### 2.7. Preparation of PTX-Loaded Poly (Mal-Peg- HPLA-cPt) Nanoparticles (PTX-PHPLA-cPt-NPs)

The synthesized poly (mal-Peg-HPLA-cPt) (11 mg) was weighed and dissolved in a mixture of acetone (2.8 mL) and ethanol (1 mL) by vortexing. The solution was mixed with 200 μL of PTX ethanol solution (1 mg mL^−1^) and then vortexed. The mixture was added dropwise to 40 mL DIW (in a 100 mL beaker) containing 1 mL acetone while stirring at RT to form nanoparticles by the nanoprecipitation method. Furthermore, this mixture was stirred for 4 h at RT to evaporate the organic solvent. The mixture was collected into a centrifugation tube and centrifuged for 15 min at 11,000 rpm at 4 °C. A total of 4 mL of DIW water was added to the pellet, and after 5× dilution and filtration, the size and zeta potential were measured using a dynamic light scattering (DLS) instrument (Brookhaven 90 plus particle size analyzer). The aliquot was stored at −80 °C for 4 h and lyophilized for 2 days by freeze-drying.

### 2.8. Conjugation of Antibody to the Nanoparticle via Thiol-Maleimide Chemistry

To conjugate the targeting ligand monoclonal antibody to the surface of PTX-PHPLA-cPt-NPs, mAb (cetuximab) was reduced with Tris(2-carboxyethyl) phosphine hydrochloride (TCEP), producing a sulfhydryl group. To obtain the thiolated antibody, cetuximab (CTX) was mixed with TCEP (2.75 mol excess) and incubated for 15 min at RT [41]. Furthermore, to prepare antibody-decorated nanoparticles, a 1:100 molar ratio of thiolated antibody to maleimide moieties in polymeric nanoparticles (PTX-PHPLA-cPt-NPs) was reacted in an aqueous solution for 2 h at RT via thiol-maleimide chemistry [42,43]. Free-activated maleimide groups were blocked using 0.5 μL of 2-mercaptoethanol [44]. To remove any excess CTX from the conjugated antibody, the reaction mixture was centrifuged at 11,000 rpm for 40 min at 4 °C. After collecting the pellet, it was resuspended in DIW and freeze-dried for 2 days using a lyophilizer (Labconco, Kansas City, MO, USA). A known amount of dried nanoparticles was utilized in an in vitro release study to determine the drug content of the nanoparticles. Antibody attachment was analyzed by the bicinchoninic acid (BCA) protein assay kit (Thermo Fisher Scientific Inc., Rockford, IL, USA) and UV-Vis spectrophotometer [45].

### 2.9. Measurement of Hydrodynamic Size, Zeta Potential, and Morphology

Nanoparticle hydrodynamic diameter and zeta potential were measured by dynamic light scattering (DLS) using a zeta-potential and particle size analyzer (Brookhaven 90 plus, Nashua, NH, USA) after 5 times dilution and filtration with a 0.45 µm syringe filter. The morphology of each nanostructure was also observed by scanning electron microscope (SEM). Nanoparticle formulations were placed on carbon stubs, dried under vacuum overnight, and observed by SEM HEIOS NANOLab 660, Thermo Scientific (Hillsboro, OR, USA).

### 2.10. In Vitro Drug Availability Study

An in vitro drug release study was performed using previously reported methods with minor modifications [46,47]. CTX-PTX@PHPLA-cPt-NPs (2 mg) solution (2 mL in DIW, 1 mg mL^−1^ of NPs) was prepared and loaded into pre-hydrated dialysis membrane tubing with a molecular weight cut-off of 12–14 kDa (Spectra/Por™ 4 RC, Spectrum Chemical Mfg. Corp., New Brunswick, NJ, USA). The tube with the sample was immersed in 10 mL of acetate buffer (pH 5) containing 0.2% (*w v*^−1^) Tween 80 in a 15 mL conical tube and incubated at 37 °C. The tubes were placed in a Labquake^®^ shaker capable of 360° rotation maintained at 37 °C in an endotherm laboratory oven (Fisher Scientific, USA). A total of 2 mL of the dissolution medium was collected at different time points (0, 1, 3, 6, 24, 72, 168, 240, and 336 h), and an equivalent volume of fresh medium was added to maintain a constant volume. HPLC and atomic absorption spectrometer (AAS) analyses were used to quantify PTX and cPt released, respectively [48,49]. To prepare an HPLC sample, collected samples (500 μL) at each time point were mixed with the same volume of acetonitrile and vortexed. In the case of AAS sample preparation, 1.5 mL of the sample was mixed with 1.5 mL of DIW and poured into an AAS tube. The drug content was calculated using the calibration curves created from pure drug samples.

### 2.11. In Vitro Antiproliferation Study

The triple-negative human breast adenocarcinoma cell line MDA-MB-231 (ATCC) was cultured in L-15 medium supplemented with 1% penicillin-streptomycin and 10% fetal bovine serum (Sigma) as media at 37 °C without CO_2_. The MDA-MB231 cells (5 × 10^3^ per well) were grown overnight in 96-well plates in 100 μL of L-15 medium. Following this, the culture medium was replaced with a medium (50 μL) containing a serial dilution of each formulation at 0.032–20 μM of PTX. After incubating for 24 h, 48 h, and 96 h, 100 µL of CellTiterGlo solution was added to each well, and the plates were shaken for 2 min, followed by 10 min of incubation. Then, the luminescence was measured using a CLARIOstar microplate reader (BMG LABTECH, Ortenberg, Germany). Each group consisted of four wells; the means of their values were used as the measured values. The mean ± SD values were used for the expression of data.

### 2.12. In Vitro Cellular Uptake

An in vitro cell uptake study was also performed using flow cytometry with rhodamine 123 (Rhd)-loaded nanoparticles. Human adenocarcinoma MDA-MB-231 cells (2.5 × 10^5^ per well) were seeded in 6-well plates in L-15 medium supplemented with 10% fetal bovine serum followed by incubation overnight to attach. After that, the cells were exposed to Rhd-loaded polymeric nanoparticles or CTX-Rhd-polymer nanoparticle suspension at a Rhd concentration of 1.5 μM for 3 h at 37 °C. Then, the cells were washed twice with cold PBS to terminate the uptake, and the nanoparticles adsorbed on the cell membrane were removed. Following that, the cells were harvested using 0.25 mL of 0.25% trypsin, ethylenediamine tetraacetic acid (EDTA) with the medium, and centrifuged. Furthermore, the cells were washed twice with PBS-2% FBS (fetal bovine serum) and resuspended in 0.4 mL of PBS-2% FBS containing propidium iodide (PI, 0.1 μg mL^−1^). The cells were examined through flow cytometry analysis using the Cytoflex S flow cytometer (Beckman Coulter, Brea, CA, USA), and the data were assessed using Floreada.io software (https://floreada.io/).

An in vitro cellular uptake study was also performed using previously reported methods with minor modifications [48]. Human adenocarcinoma MDA-MB-231 cells (2 × 10^5^ per well) were seeded in 12-well plates in L-15 medium supplemented with 10% fetal bovine serum followed by incubation overnight to attach. After that, the cells were exposed to cPt in DMSO (0.1% in water) or PTX/cPt polymeric nanoparticles or CTX-PTX-cPt-polymeric nanoparticle suspension at a cPt concentration of 1.5 μM for 3 h at 37 °C. At predetermined time intervals, the cells were washed three times with ice-cold phosphate-buffered saline (PBS) to terminate the uptake and remove the nanoparticles that adsorbed on the cell membrane. Then, the cells were harvested using 0.25 mL of 0.25% trypsin, ethylenediamine tetraacetic acid (EDTA) with the medium, and centrifuged. To the cell pellet, 1 mL of DIW was added, and the cell pellet was subjected to a probe sonicator for 30 s (5 s on and 5 s off) with an output power of 30% amp in an ice bath. After sonication, 1 mL of cell lysate was centrifuged at 20,000 rcf at 4 °C for 5 min, and the supernatant was diluted with DIW till 2.5 mL. The content of cPt in the aliquot was analyzed by AAS. The protein content was determined using the bicinchoninic acid (BCA) protein assay kit (Thermo Fisher Scientific Inc., Rockford, IL, USA). Cellular accumulation of cisplatin was normalized with a reset to total protein content that is in cell lysate. The amount of cisplatin found was divided by the total protein in the cell lysate.

### 2.13. Intracellular Localization of Nanoparticles by Confocal Microscopy

An intracellular localization study was carried out according to previously reported methodologies [50,51]. Briefly, the triple-negative human breast adenocarcinoma cells (MDA-MB-231) were seeded onto 6-well plates inserted with coverslip at 6 × 10^5^ cells per well in 2 mL of L-15 medium supplemented with 10% fetal bovine serum, followed by incubation overnight to attach. Three groups of wells were investigated as follows: 10 μg mL^−1^ of free Rhd, Rhd-loaded plain NPs, and CTX-Rhd-loaded NPs were added to each group of well and incubated with the cells for 1, 6, and 24 h at 37 °C. To visualize cell internalization, the cells were washed twice with cold PBS, fixed with 4% paraformaldehyde (2 mL) for 5 min, and washed with cold PBS once. Then, the cells were further incubated with Hoechst R 33342 (5 μg/mL) nucleus stain and then CellMaskTM deep red plasma membrane stain (4 μg mL^−1^) for 1 min. Then the slides were rinsed with PBS once. Finally, the slides were mounted with fluoromount (Sigma, St. Louis, MI, USA) on glass slides and dried overnight at RT. The slides were observed with a confocal laser scanning microscope (CLSM 510; Carl Zeiss AG, Jena, Germany) using a 40 × 1.3 NA Plan-Apochromat oil immersion objective and a multitrack configuration. The Hoechst^R^ 33342, Rhd-loaded nanoparticles, and CellMask^TM^ deep red stain signals were collected by using a BP 385–470 nm filter, 505–550 nm filter, and LP 650 nm filter after excitation with the 364-, 488-, and 633-nm laser lines, respectively. Images (512 × 512 pixels) were acquired with a line average of four by using the Zeiss AIM software (ZEN 3.8).

### 2.14. Cell Binding Assay of CTX-Nanoparticles by Flow Cytometry Analysis

The cells (MDA-MB-231) were harvested using trypsin-EDTA and counted. The cells were washed with PBS supplemented with 2% FBS twice. The washed cells were transferred into four 15 mL conical tubes, each containing 2.5 × 10^5^ cells mL^−1^ (1 mL per tube). Cells were incubated with unconjugated (pure) antibody (CTX) or nanoparticle-conjugated antibody (CTX-NPs) (20 μg mL^−1^) in PBS-2% FBS for 1 h at 4 °C under gentle agitation. In order to establish non-binding antibody control, a separate tube was incubated with human IgG1 isotype control (20 μg mL^−1^). Then, the cells were washed twice with PBS-2% FBS and incubated with FITC-conjugated anti-human IgG1-Fc antibody (20 μg mL^−1^) diluted with PBS-2% FBS in the dark, for 30 min at 4 °C under gentle agitation. Furthermore, the cells were washed twice with PBS-2% FBS and resuspended in 0.4 mL of PBS-2% FBS containing propidium iodide (PI, 0.1 μg mL^−1^). The cells were then subjected to flow cytometer analysis using the Cytoflex S flow cytometry (Beckman Coulter, Brea, CA, USA), and the data were analyzed using Floreada.io software (https://floreada.io/).

### 2.15. Statistical Analysis

All of the studies were carried out in triplicate, and the results were expressed as mean ± standard deviation (S.D.). The statistical significance of the data was analyzed by Student’s *t*-test and one-way ANOVA. In all cases, *p* < 0.05 or *p* < 0.01 was considered to be statistically significant.

## 3. Results

### 3.1. Cisplatin Modification

**Figure 1:**
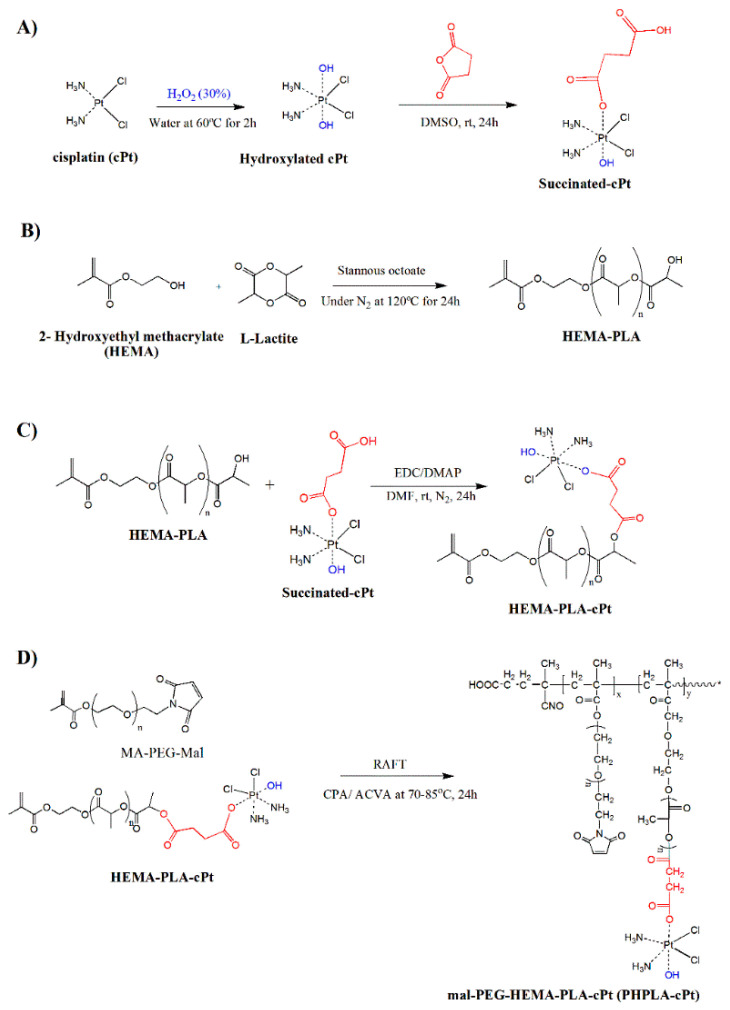
Synthesis scheme of cisplatin–polymer conjugate. (**A**) Succinated cisplatin synthesis from hydroxylated cisplatin and succinic anhydride at 1:1 molar ratio. Hydroxylated cisplatin was obtained by treatment of hydrogen peroxide; (**B**) HEMA–PLA synthesis by ring–opening polymerization of L–lactide with 2–Hedroxyethyl methacrylate at 5.6:1 molar ratio; (**C**) synthesis of HEMA–PL–cPt conjugate from hydroxyethyl methacrylate polylactide and succinated cisplatin at 1:1 molar ratio using carbodiimde reaction; (**D**) RAFT polymerization of MA–PEG–mal and HEMA–PLA–cPt at 10:1 molar ratio to obtain mal-PEG-HEMA-PLA-cPt (PHPLA–cPt).

**Figure 2:**
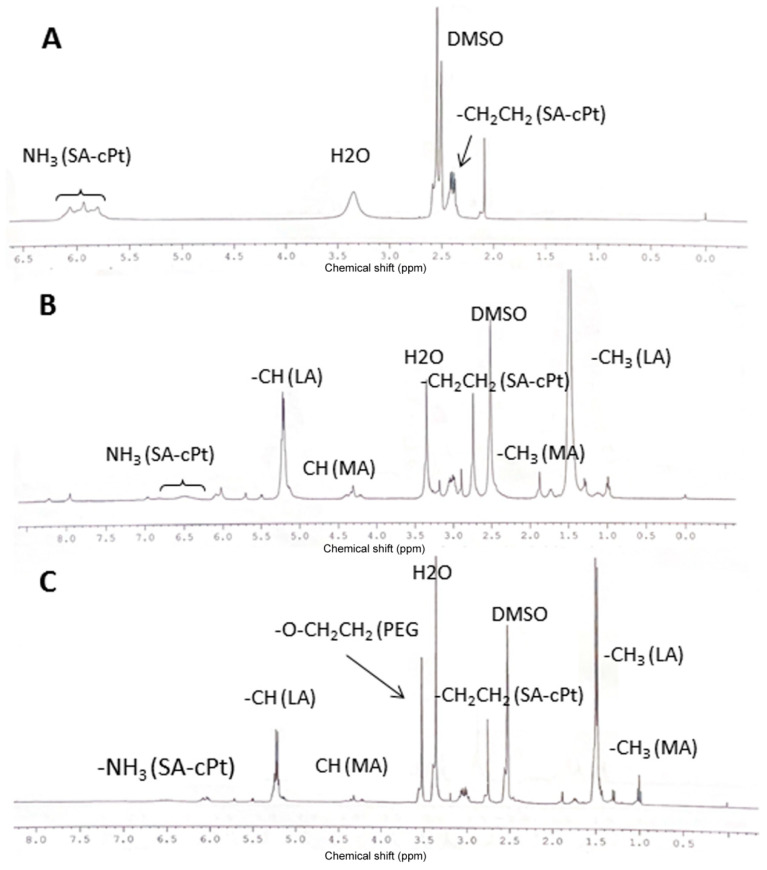
Proton NMR spectra of SA-cPt conjugate (**A**), HPLA-cPt conjugate (**B**), and PHPLA-cPt copolymer (**C**). DMSO-d6 is used to dissolve all samples before measuring them by NMR. LA represents Lactate, MA represents methacrylate moiety, and SA represents succinic acid residue.

In order to covalently attach cisplatin to the polymer backbone, it was modified according to the method described in the literature (Figure 1A) [35,36]. First, the cisplatin was oxidized using an excess amount of hydrogen peroxide and heating at 70–80 °C. The yield of oxidized cisplatin (hydroxylated cisplatin) was over 90%, and yellow powder was produced. Oxidation was confirmed by FT-IR analysis, and specific signals at 1041 cm^−1^ for Pt-OH and 3000 cm^−1^ and 3473 cm^−1^ for hydroxyl groups were observed as compared to unmodified cisplatin (Figure S1). Furthermore, the oxidized cisplatin was reacted with succinic anhydride in DMSO with a one-to-one molar ratio to obtain one-side succinated cisplatin overnight at RT [35,36]. The reaction product was a yellowish powder with a yield of 80.1%. The succinate-cisplatin was analyzed by proton NMR and FT-IR. Proton NMR data showed peaks at 5.808–6.068 ppm for NH_3_ of the cisplatin moiety and peaks at 2.38–2.412 ppm for -CH_2_-CH_2_- of succinate, respectively (Figure 2A). The FT-IR spectroscopy presented signals at 3200–3400 cm^−1^ for NH_3_ and OH and at 1710 cm^−1^ for N-Pt of cPt-SA, respectively (Figure S4B).

### 3.2. Synthesis of HEMA-PLA and HEMA-PLA-Cisplatin Conjugate

**Figure 3:**
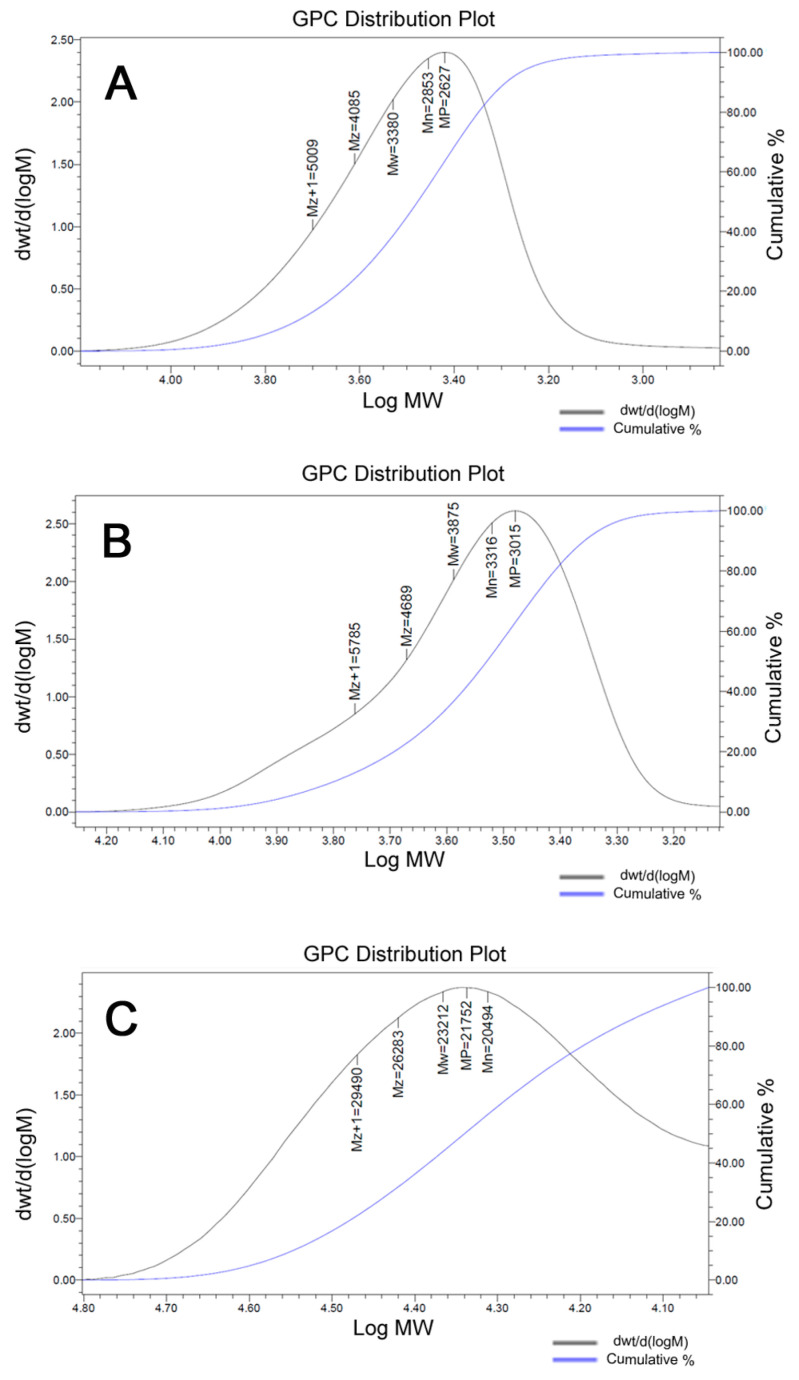
GPC results for macromonomers and polymers. (**A**) represents HEMA-PLA macromonomer in THF, (**B**) represents HEMA-PLA-cPt conjugate, and (**C**) represents PHPLA-cPt polymer.

HEMA-PLA macromonomer synthesis was completed according to the previously reported methodology [37]. L-Lactide (LA) was polymerized by a ring-opening polymerization reaction with HEMA in the presence of stannous octoate as a catalyst under nitrogen at 120 °C (Figure 1B). The reaction product, HEMA-PLA, was purified by recrystallization and filtration methods. The formation of HEMA-PLA was confirmed by proton NMR and FT-IR spectrometers (Figures S2 and S3). The GPC results showed that the average molecular weight (MP) of the macromonomer was 2627 Da with a PDI of 1.18 (Figure 3A), which corresponds to the estimated number average molecular weight from proton NMR data.

Furthermore, HEMA-PLA was reacted with succinate-cisplatin conjugate (SA-cPt) through a classic carbodiimide reaction between the hydroxyl group of HEMA-PLA and the carboxyl group of SA-cPt overnight (Figure 1C). After recrystallization, washing with diethyl ether, and filtering the reaction product (HPLA-cPt), a yellowish powder was obtained. Proton NMR, FT-IR, and GPC were used to demonstrate the formation of the product. Some specific peaks in the proton NMR, in addition to the peaks of HEMA and LA, were found at 6.505–6.539 ppm, which correspond to the NH_3_ group of the cisplatin moiety in the HPLA-cPt conjugate (Figure 2B).

In FT-IR spectra (Figure S4C), a broad peak at 3000–3500 cm^−1^ represented O-H stretching hydroxyl groups of HPLA, while sharp peaks at 1757 cm^−1^ and 1090 cm^−1^ represented –C=O and –C-O-C- stretching of carbonyl and ether backbones of HPLA. Additionally, a small peak at 1645 cm^−1^ represented the N-Pt stretching of the cPt-SA moiety. The GPC data revealed that the MP of HPLA-cPt was 3015 Da, with a PDI of 1.17, indicating that the cPt-SA moiety was attached to the macromonomer (Figure 3B).

### 3.3. Synthesis of Poly (Mal-HPLA-cPt) by RAFT Polymerization

Cisplatin-attached macromonomer (HPLA-cPt) was reacted with mal-PEG-MA through RAFT polymerization reaction in the presence of CPA catalyst and a mixture of ethanol and DMF as a solvent under nitrogen overnight. The reaction product was collected through filtration and washed with DE (diethyl ether), followed by vacuum drying. The resulting solid polymer was characterized by proton NMR in DMSO-d6, FT-IR, and GPC in THF/toluene. The formation of the polymer was confirmed by proton NMR revealing specific signals at 1.472–1.49 ppm for the lactide moiety and 4.22–4.35 ppm for the methacrylate moiety (Figure 2C). In FT-IR spectra (Figure S4A), a broad peak at 3000–3500 cm^−1^ represented O-H stretching hydroxyl groups of HPLA, while sharp peaks at 1757 cm^−1^ and 1090 cm^−1^ represented –C=O and –C-O-C- stretching of carbonyl and ether backbones of HPLA. Additionally, a small peak at 1642 cm^−1^ represented the N-Pt stretching of the cPt-SA moiety. The GPC results showed that the MP of poly (mal-HPLA-cPt) was 21,752 Da with a PDI of 1.18, which indicates that the polymer consists of around seven macromonomers (Figure 3C).

### 3.4. Synthesis of PTX-Loaded Mal-PEG-HPLA-cPt Nanoparticles (PTX-PHPLA-cPt NPs) and CTX-Attached PTX-PHPLA-cPt NPs

**Figure 4:**
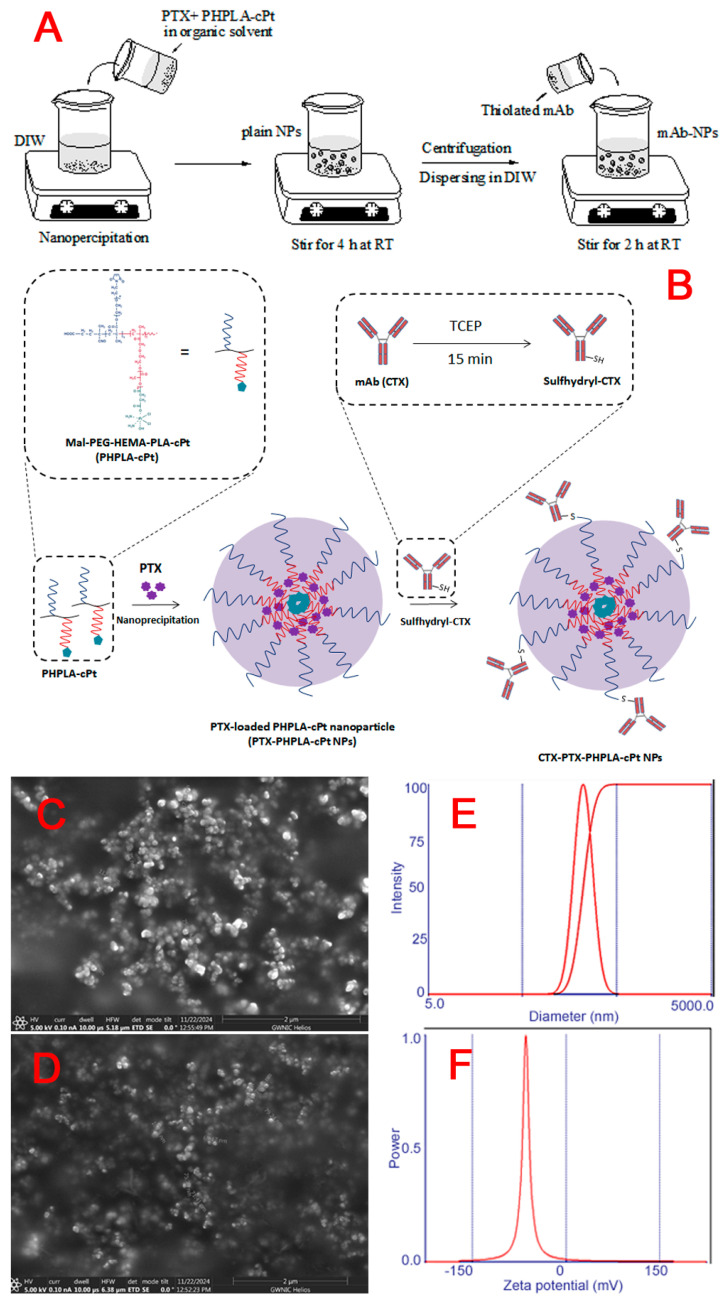
(**A**) Fabrication of nanoparticles using nanoprecipitation method. (**B**) Scheme of preparation of nanoparticles containing PTX and cisplatin and attachment of cetuximab (CTX) to their surfaces. The upper left panel presents the modeling of Mal-PEG-HEMA-PLA-cPt copolymer; the upper right panel describes how TCEP can reduce disulfide linkage in mAb. Size and morphology of CTX-PTX@PHPLA-cPt NPs: (**C**,**D**) SEM images of the CTX-NPs and p-NPs, (**E**) mean diameter, and (**F**). zeta potential of the CTX-NPs by DLS.

Following the synthesis of PHPLA-cPt conjugate, we prepared nanoparticles in the presence of PTX, a hydrophobic anticancer drug, using a nanoprecipitation method (Figure 4A).

To prepare the nanoparticle, the polymer conjugate (containing cisplatin) and PTX were dissolved in acetone and ethanol at a 2.5:1 ratio, and the mixture was added dropwise to DIW containing 2.5% acetone. The mixture was stirred for 4 h at RT to evaporate the organic solvent. Furthermore, the aqueous solution was transferred into the centrifugation tube and was centrifuged to separate the formed nanoparticles. The pellet was dispersed in DIW (5 mL), and the size and surface charge were measured by a DLS instrument (Brookhaven 90 plus particle size analyzer) after being diluted five times with DIW. As presented in Table 1 and Figure 4, the average size of plain NPs was 161.6 ± 3.2, with a PDI of 0.209, and the surface charge was found to be −12.41 ± 0.37, indicating that they are suitable for medical application.

To conjugate mAb (cetuximab; CTX), a known amount of the obtained plain nanoparticles (PTX-PHPLA-cPt NPs) was dispersed in DIW and mixed with thiolated CTX. In the case of thiolation, CTX was reacted with TCEP at a ratio of 17 to 1, which can partially cleave the disulfide bond in the hinge region of the antibody, for 15 min [41] (Figure 4A). Furthermore, the reduced CTX was mixed with the nanoparticle suspension and stirred at RT for 2 h to react. A small amount of 2-mercaptoethanol was added after the reaction to neutralize the free maleimide group on the NPs. Then, the mixture was centrifuged to separate the formed nanoparticles. After dispersing the pellet in DIW (5 mL), the size and surface charge were measured using a DLS instrument after diluting five times with DIW. The average size of CTX-NPs was 198.7 ± 1.3, with a PDI of 0.047, and the surface charge was found to be −41.32 ± 1, indicating that the CTX is attached to the surface of the NPs (Table 1 and Figure 4). The suspension with 5% trehalose was frozen at −20 °C overnight and freeze-dried for 2 days. SEM images of CTX-NPs and p-NPs are displayed in Figure 4B,C. The SEM images (Figure 4B) show CTX-NPs with a homogeneous size and spherical morphology.

To analyze the presence of CTX on the surface of NPs, we used a BCA kit that can react with a protein molecule and change the color of the solution from blue to purple. The optical absorption was measured using a UV-Vis spectrophotometer (Figure S5). As shown in Figure S5, the absorption peaks appeared at 550 nm for the mixture of CTX and BCA reagent and the CTX-NPs and BCA reagent, while no absorption at 550 nm was found from the mixture of plain NPs and BCA reagent; it was only found from the BCA reagent. This indicates that the CTX antibody is attached to the surface of NPs.

### 3.5. In Vitro Drug Release Study

An in vitro drug release study was conducted using a dialysis membrane for 14 days. As a release medium, an acetate buffer with pH 5 containing 0.2% Tween 80 was chosen to mimic the extracellular environment of tumor tissue. The released drugs were analyzed using RP-HPLC and AAS methods.

**Figure 5:**
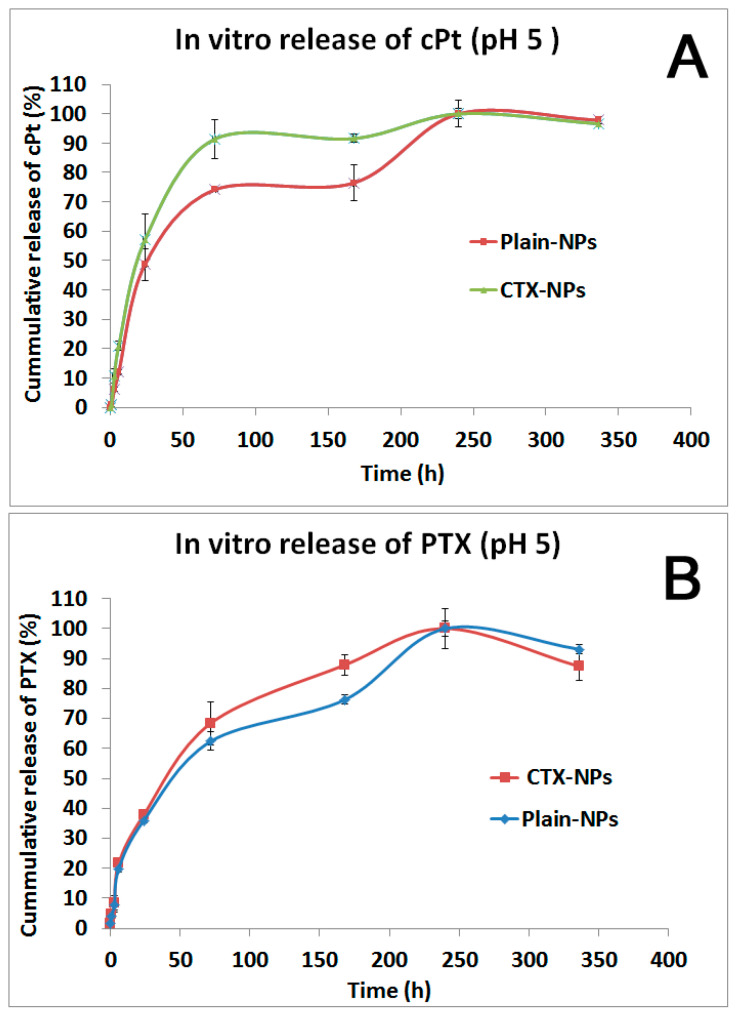
In vitro release study. (**A**) Cumulative release profile of cisplatin (cPt) from both plain NPs and CTX-attached NPs at pH 5.0; (**B**) cumulative release profile of paclitaxel (PTX) from both plain NPs and CTX-attached NPs at pH 5.0.

As shown in Figure 5A,B, PTX and cPt were gradually released up until the 10th day after incubation at 37 °C. The release profile of drugs from both plain NPs and CTX-NPs had a similar pattern. In the case of CTX-NPs, the drugs were released slightly faster than in plain NPs. PTX release by 24 h was 37.8% and 35.9% for CTX-NPs and plain NPs, respectively. Meanwhile, cPt released by the same time point was 57.02% and 48.6% from CTX-NPs and plain NPs, respectively. The drug content in the nanoparticles was calculated based on the release profile. According to the findings, plain NPs and CTX-NPs contained 1.46% and 1.33% of PTX. On the other hand, the content of cPt was obtained at 0.63% and 0.48% in plain NPs and CTX-NPs, respectively. To enhance the drug loading in the NPs, we are planning to increase the molecular weight of the copolymer and the length of the hydrophobic moiety.

### 3.6. Cellular Uptake Study by Flow Cytometry and AAS

**Figure 6:**
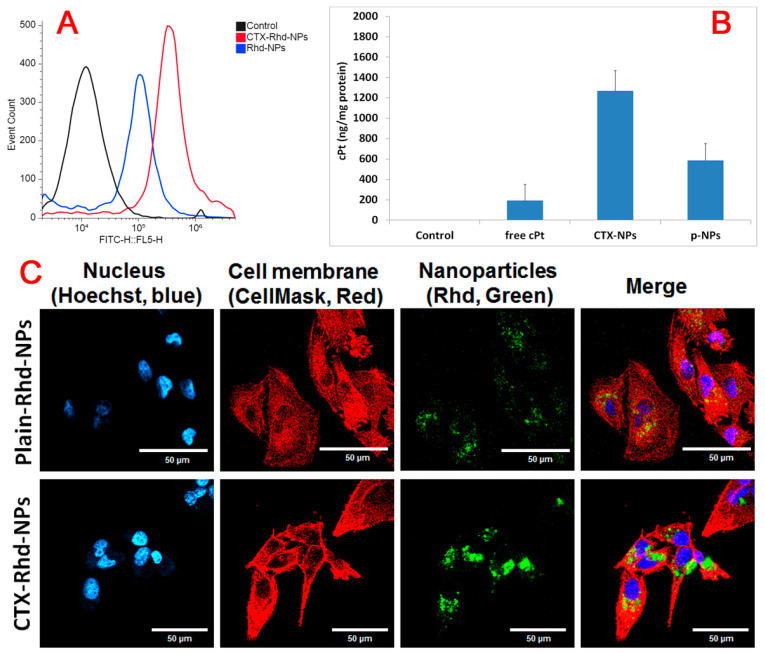
(**A**) After 3 h of incubation at 37 °C, the cells were analyzed by flow cytometer to characterize the uptake of Rhd-NPs and CTX-Rhd-NPs. Here, rhodamine (Rhd) was applied as a payload that has a fluorescence signal instead of a PTX molecule. (**B**) After 3 h of incubation at 37 °C, cellular uptake of cisplatin was measured by AAS. The cisplatin content was normalized based on the protein amount in the cells using BCA assay [48]. ANOVA table shows that there is a statistically significant difference among the three treatment groups (free cisplatin (free cPt), plain nanoparticles (p-NP), and monoclonal antibody conjugated nanoparticles (CTX-NP) (*p* < 0.01). Treatment contrasts p-NP versus CTX-NP (*p* < 0.01); free cPt versus CTX-NP (*p* < 0.01; and free cPt versus p-NP (*p* < 0.01). (**C**) Cellular uptake of plain Rhd-NPs and CTX-Rhd-NPs by confocal microscope after 48 h of incubation in MDA-MB-231 cells. Hoechst (blue) indicates the nucleus, CellMask Red (red) indicates the cell membrane, and Rhd (green) presents rhodamine 123 as a payload.

Flow cytometry was performed to analyze cell uptake of Rhd-loaded plain NPs (plain Rhd-NPs) and CTX-NPs (CTX-Rhd-NPs) in the MDA-MB-231 cell line. As shown in Figure 6A, after 3 h of incubation, CTX-Rhd-NPs were highly taken up by the cells compared to plain Rhd-NPs, which suggests that CTX enhanced the internalization of NPs by the cells.

A cellular uptake study of free cPt, PTX-loaded cPt-NPs (plain NPs), and CTX-attached PTX-loaded cPt-NPs (CTX-NPs) in MDA-MB-231 cells was conducted. cPt content in the cells at 3 h after incubation was measured by an AAS spectrometer. In order to measure the amount of cPt, cells were harvested and lysed using freeze–thaw cycling and probe sonication. Furthermore, the drug content was analyzed in the separated aliquot from the cell lysate. In the remaining lysate, the protein content was measured using a BCA assay and applied to optimize the drug content in the cell. As shown in Figure 6B, at 3 h, antibody-attached NPs were highly taken up by the cells as compared to free cPt and plain NPs. The amount of cPt in CTX-NP-treated cells after 3 h of incubation was 6.6- and 2.2-fold higher than that in free cPt and plain NP-treated cells, respectively. These results indicate that CTX-mediated internalization of NPs has occurred due to overexpression of EGFR in the MDA-MB-231 TNBC cell line. Also, these results are consistent with the results of the cytotoxicity study.

### 3.7. Confocal Microscope Study

**Figure 7:**
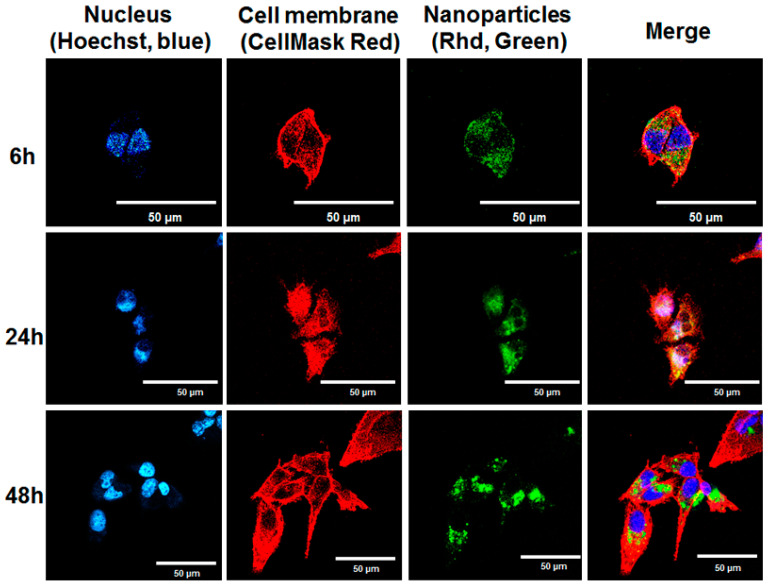
Cellular uptake of CTX-Rhd-NPs by confocal microscope after incubation for 6 h, 24 h, and 48 h in MDA-MB-231 cells. Hoechst (blue) indicates the nucleus, CellMask Red (red) indicates the cell membrane, and Rhd (green) presents rhodamine 123 as a payload.

A confocal microscope was used to conduct an intracellular uptake study of Rhd-NPs and CTX-Rhd-NPs in MDA-MB-231 cells. Rhd was substituted for a drug molecule in order to make it a fluorescent NP. Figure 6C presents a comparison of intracellular uptake between plain Rhd-NPs and antibody-attached NPs (CTX-Rhd-NPs) after 48 h of incubation. Here, the nucleus was stained with Hoechst, and the cell membrane was stained with CellMask Red. The Rhd is green in color. The results showed that, after 48 h of incubation with CTX-Rhd-NPs, a significantly higher amount of Rhd was taken up by the cells as compared to that of plain Rhd-NPs, indicating that EGFR-mediated internalization of CTX-attached NPs occurred. Additionally, as shown in Figure 7, the cellular uptake of CTX-Rhd-NPs was time-dependent from 6 h to 48 h. The green signal of Rhd was stronger after 48 h of incubation compared to that after 6 h and 24 h, indicating that Rhd was released more after 48 h.

### 3.8. Cell Binding of CTX-NPs and Cytotoxicity on MDA-MB-231 Cells

**Figure 8:**
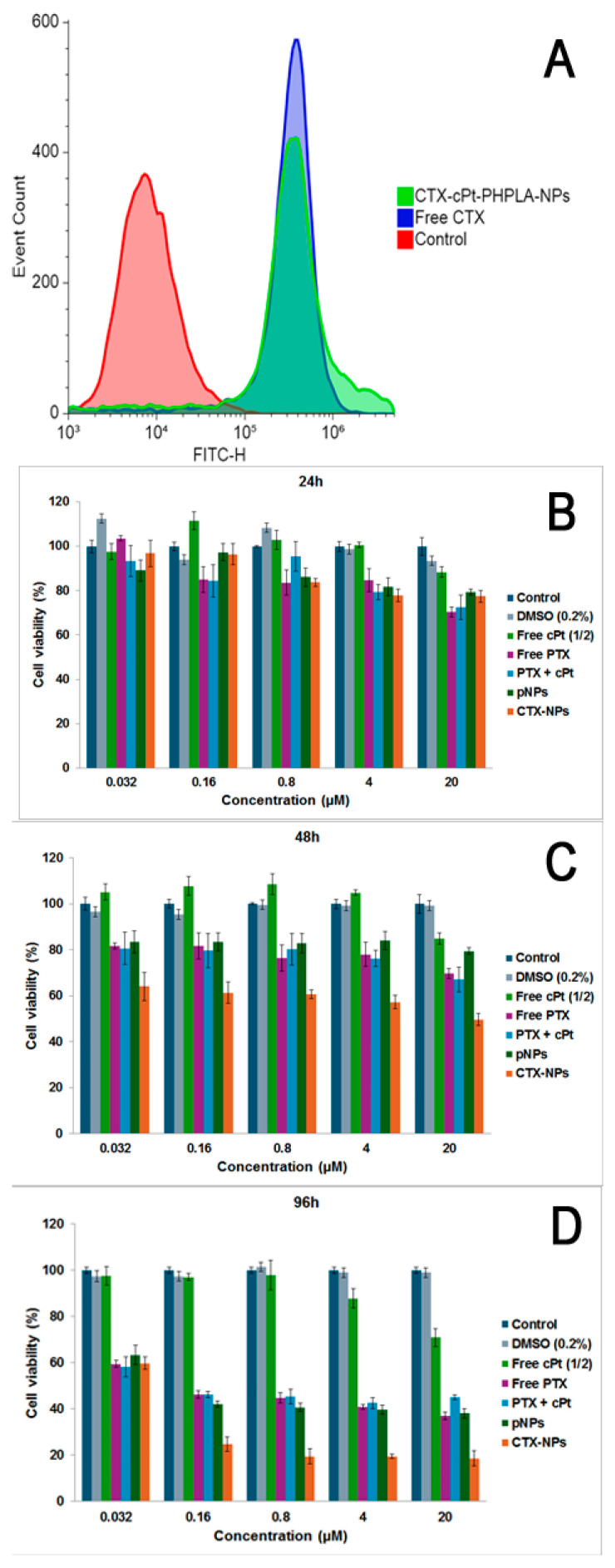
(**A**). Cell binding of CTX-NPs on MDA-MB-231 cell line by FACS. All groups were stained with PI, and FITC-labeled IgG secondary antibody was added to the groups treated with CTX-NPs (CTX-PTX-PHPLA-cPt-NPs) and free CTX. Cytotoxicity study by CellTiterGlo luminescence assay: Cell cytotoxicity results at 24 h (**B**), 48 h (**C**), and 96 h (**D**) after treatment with free cPt and PTX, combination of cPt and PTX, drug-loaded plain nanoparticle (p-NPs), and antibody-attached nanoparticles (CTX-NPs) against MDA-MB-231 cell line at 37 °C were shown. The IC_50_ values of CTX-NPs, p-NPs, and PTX + cPt after 96 h of incubation were 0.1 µM, 0.49 µM, and 0.57 µM, respectively.

To analyze the binding ability of the CTX antibody after attachment to the NPs on TNBC cells, we conducted a flow cytometer analysis after 1 h of incubation with CTX-NPs or free CTX. Here, a secondary antibody labeled with FITC was applied to determine the bound CTX on the surface of MDA-MB-231 cells. Also, propidium iodide (PI) was added to stain the nucleus to make it detectable for flow cytometer analysis. As shown in Figure 8A, free CTX and CTX-NPs have strong binding to cells compared to both the control group and the group treated with human IgG antibodies. The results demonstrated that CTX attached to the NPs still retained its binding ability to cells that express EGF receptors.

To determine whether the nanoparticles are potent against TNBC cells, we conducted a cell viability study using CellTiterGlo, which detects ATP in viable cells by measuring luminescence after treatment with drug-containing nanoparticles in the MDA-MB-231 cell line. The cell viability was measured in the cells after incubation with free drugs, plain NPs, and CTX-NPs for 24 h, 48 h, and 96 h. Figure 8B shows that cell viability after 24 h of treatment was over 80% for all cells, including those treated with nanoparticles, indicating that very few cells were killed.

After 48 h of incubation (Figure 8C), 64% and 49.6% of cell viability was obtained at 0.032 µM and 20 µM of PTX (0.016 µM and 10 µM of cPt) of CTX-NPs, which was the most cytotoxic group compared to other groups. As presented in Figure 8D, 24.7%, and 18.6% of cell viability were found in cells treated with 0.16 µM and 20 µM of PTX (0.08 µM and 10 µM of cPt) of CTX-NPs, respectively, after 96 h of incubation, which was higher cytotoxicity against the MDA-MB-231 cell line compared to other groups, including plain NPs and free drugs. The IC_50_ of CTX-NPs after 96 h of incubation was 0.1 µM, which was significantly higher than those of p-NPs (0.49 µM) and PTX + cPt (0.57 µM). Our data showed that a combination of PTX and cPt was able to kill more cells, indicating the combinatorial potential of CTX-NPs.

## 4. Discussion

The purpose of this research was to develop dual-loaded multifunctional polymeric nanoparticles (containing hydrophilic (cisplatin) and hydrophobic (paclitaxel) drugs that have a strong targeting ability with respect to tumor cells (by virtue of cetuximab attached to the nanoparticle surface), specifically TNBC. TNBC is one of the cancer types that cannot be treated with currently available single-targeted therapies due to its lack of common receptors. Drug resistance is another problem. As therapeutic agents, we chose cPt and PTX, commercially available anticancer drugs, due to their high potency and wide application for TNBC treatment. First, we modified cisplatin to be able to conjugate it to a polymer backbone according to previously known approaches. Since the cPt molecule is highly water soluble, its encapsulation into the hydrophobic cores of polylactide nanoparticles is limited. Therefore, we decided to conjugate it directly to the polymer backbone by chemical bonding, which can increase drug loading and prolong drug release time. We found that the rate of release of cPt from CTX-NPs was significantly slower than that of noncovalently incorporated nanoparticles. Furthermore, a previous study showed that a cPt-attached PLA polymer nanoparticle released 80 percent of the drug within 75 h, which is similar to our data in this manuscript [52]. In the case of PTX, we preferred to incorporate it into the nanoparticles through hydrophobic interaction due to its strong hydrophobicity for the lactide hydrophobic core of the nanoparticles. To obtain a drug–copolymer conjugate, we synthesized the HEMA-PLA macromonomer that contains a hydroxyl functional group at the end. The succinated cPt molecule was attached to the hydroxyl group by the EDC/DMAP coupling reaction and formed an ester bond. The ester bond is known to be biodegradable by hydrolysis, which allows for the slow release of the drugs from the nanoparticles, as confirmed by previous studies [53,54]. Furthermore, we synthesized mal-PEG-HEMA-PLA-cPt copolymer through RAFT polymerization, known to be the promising polymerization reaction for methacrylate monomers [55]. The reaction is much simpler than conventional free radical polymerization and provides a high yield of polymer. A mean molecular weight of 22 kDa was found by GPC, and it was optimal for preparing nanoparticles. According to a literature report, a nanoparticle with a size of 237 nm was obtained by using a copolymer that has a similar molecular weight after RAFT polymerization [37]. Our aim in synthesizing the copolymers is to prepare nanoparticles through hydrophobic interaction. To attach the monoclonal antibody, a targeting ligand, to the nanoparticles, we applied a functionalized monomer for polymer synthesis. The maleimide group readily reacts with the sulfhydryl group, forming a thioether bond. The fact that mAb has disulfide links in the backbone makes it a candidate for partial cleavage by TCEP treatment, allowing it to release free sulfhydryl groups that can be used for conjugation with nanoparticles containing the maleimide moiety. We chose this conjugation pathway because it prevents the inactivation of the free amine group of mAb, which is the active part of receptor binding. The mean diameter of nanoparticles was around 190 nm, which is within the optimal range for nanoparticles injected intravenously. The use of PEG ensures that the NPs remain stable while circulating in the blood. Attaching mAb (CTX) to the NPs slightly increased their size and significantly reduced their surface charge, indicating the presence of CTX on the surface of the NPs. An in vitro release study was performed at pH 5, which is close to the pH in the tumor microenvironment. In the case of the cytotoxicity study, we observed that while increasing incubation time, CTX-NPs revealed more cell-killing efficacy, which is associated with increased drug release. Our main purpose is to deliver drug molecules via EGFR-mediated internalization by TNBC cells, using a CTX antibody that binds to the surface EGF receptor. Therefore, we demonstrated CTX binding and EGFR-mediated internalization of CTX-NPs using flow cytometry and a confocal microscope. The cell binding study showed that CTX-NPs have a strong binding capacity to MDA-MB-231 cells, just like free CTX, which indicates their ability to deliver drugs to the cells. Since we used CTX-NPs loaded with Rhd, which is a fluorescent probe, we were able to analyze the cell uptake of the NPs using a confocal microscope. When nanoparticles enter a cell via endocytosis, they are trapped within endosomes and lysosomes, which are acidic compartments that contain enzymes that can degrade their cargo [56]. However, in our case, since we used small molecule drugs, there was no effect on degradation, unlike proteins and nucleic acids, and the payloads were able to be released to the cytoplasm by diffusion following polymer degradation [57]. In addition, in acidic endolysosomes, poly-lactide nanoparticles can undergo rapid endolysosomal escape when their surface charge is reversed [58]. A cytotoxicity study showed that the cell-killing effect was enhanced with an increase in time from 24 h to 96 h, which was consistent with the drug release profile. The literature shows that the IC_50_ values of PTX and cPt in the MDA-MB-231 cell line were 0.3–5 µM and 23 μM, respectively [59,60,61]. Our data show 0.1 µM, 0.49 µM, and 0.57 µM for IC_50_ values of CTX-NPs, p-NPs, and free drugs (PTX + cPt), respectively, after 96 h of incubation. Recently, novel therapeutics including PARP inhibitors (such as olaparib and talaroparib) and immune checkpoint inhibitors (such as aterolizumab and pembrolizumab) have been approved by the FDA for clinical trials against BCRA-mutated TNBC and other TNBCs [62,63,64]. However, these therapeutics still have conventional issues, such as side effects and poor stability. Therefore, our targeted dual-loaded nanoparticle system could have better potential against TNBC. Our data suggest that these dual-loaded targeted NPs are capable of selectively delivering drugs to TNBC; consequently, we planned to pursue in vivo studies after improving drug loading.

## 5. Conclusions

Our study led to the development of polymeric multifunctional dual-loaded nanoparticles with surface-tagged monoclonal antibodies for targeted treatment of TNBC. To prepare polymeric nanoparticles, we synthesized poly(mal-PEG-HEMA-PLA) copolymer with a 22 kDa molecular weight using RAFT polymerization. We chose two anticancer drugs (cPt and PTX) and loaded them into polymeric NPs using a covalent linkage and hydrophobic interaction, respectively. To actively deliver the NPs to the TNBC cells, CTX (mAb), a ligand for EGFR, was attached to the surface of the NPs by a thioether linkage. We obtained CTX-NPs that contain both cPt and PTX, with a diameter of around 190 nm. Furthermore, it was demonstrated that the CTX-NPs exhibit slow drug release for up to 10 days at low pH and efficient uptake by TNBC cells, owing to EFGR receptor-mediated internalization. Cytotoxicity study showed that CTX-NPs have higher efficacy against TNBC cells (MDA-MB-231), compared to plain NPs and free anticancer drugs. Our study confirmed that CTX-NPs possess excellent in vitro properties and are a promising candidate for future in vivo studies using a tumor model.

## Figures and Tables

**Table 1:** Characterization of p-NPs and CTX-NPs.

Nanoparticle	Mean Diameter (nm)	Polydispersity Index (PDI)	Zeta Potential (mV)
Plain PTX-PHPLA-cPt-NPs (p-NPs)	161.6 ± 3.2	0.206 ± 0.007	−12.41 ± 0.37
CTX-PTX-PHPLA-cPt-NPs (CTX-NPs)	198.7 ± 1.3	0.047 ± 0.004	−41.32 ± 1

## Data Availability

Data are contained within the article and Supplementary Materials.

## Supplementary Materials

The following supporting information can be downloaded at: https://www.mdpi.com/article/10.3390/pharmaceutics17040425/s1, Figure S1: FTIR spectra of free cPt and oxidized cPt (Hydroxylated cPt); Figure S2: FTIR spectrum of HEMA-PLA macromonomer; Figure S3: Proton NMR spectrum of HEMA-PLA; Figure S4: FTIR spectra of PHPLA-cPt copolymer (A), SA-cPt conjugate (B), and HPLA-cPt conjugate (C). FTIR was obtained through the use of powder samples; Figure S5: Confirmation of attachment of antibody (CTX) to the nanoparticles using BCA reagent by UV-spectrophotometer.
