# Supplementary material for: Development of Multifunctional Targeted Dual-Loaded Polymeric Nanoparticles for Triple-Negative Breast Cancer Treatment
Source: Pharmaceutics. 2025 Mar 27;17(4):425. doi: 10.3390/pharmaceutics17040425 (PMC12030066; doi:10.3390/pharmaceutics17040425)
Supplement: Supplementary file 1 [file pharmaceutics-17-00425-s001.zip › pharmaceutics-3538692-supplementary.pdf]

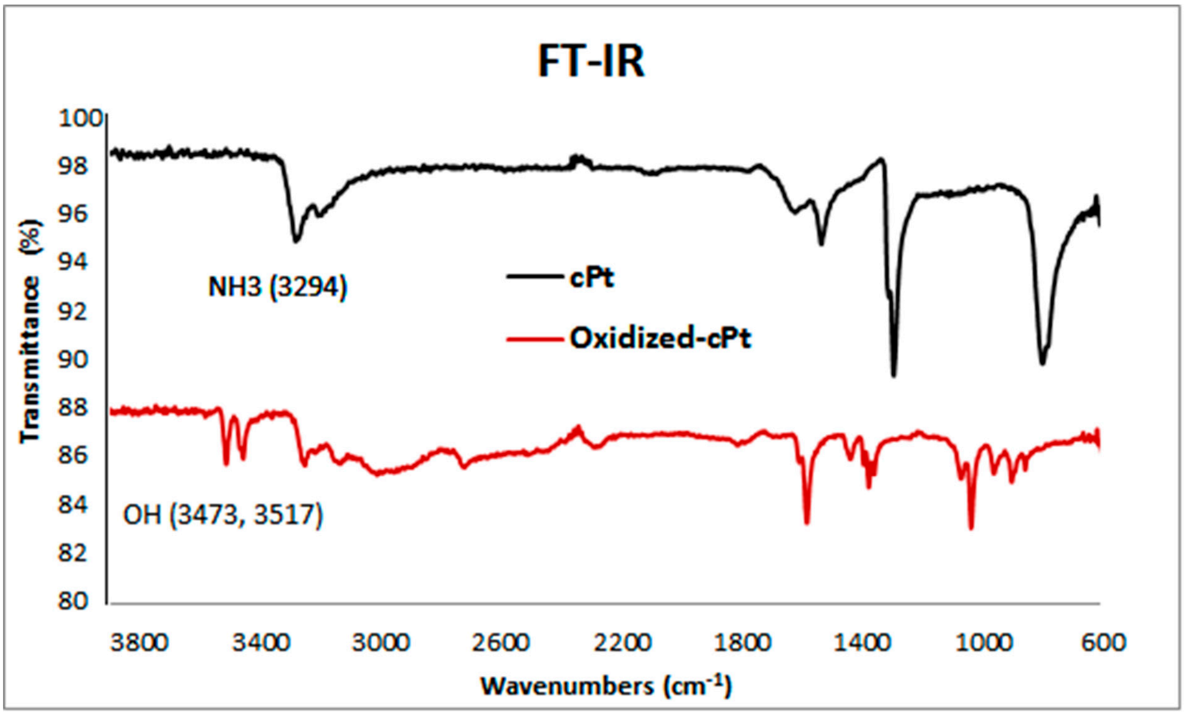

Figure S1. FTIR spectra of free cPt and oxidized cPt (Hydroxylated cPt).

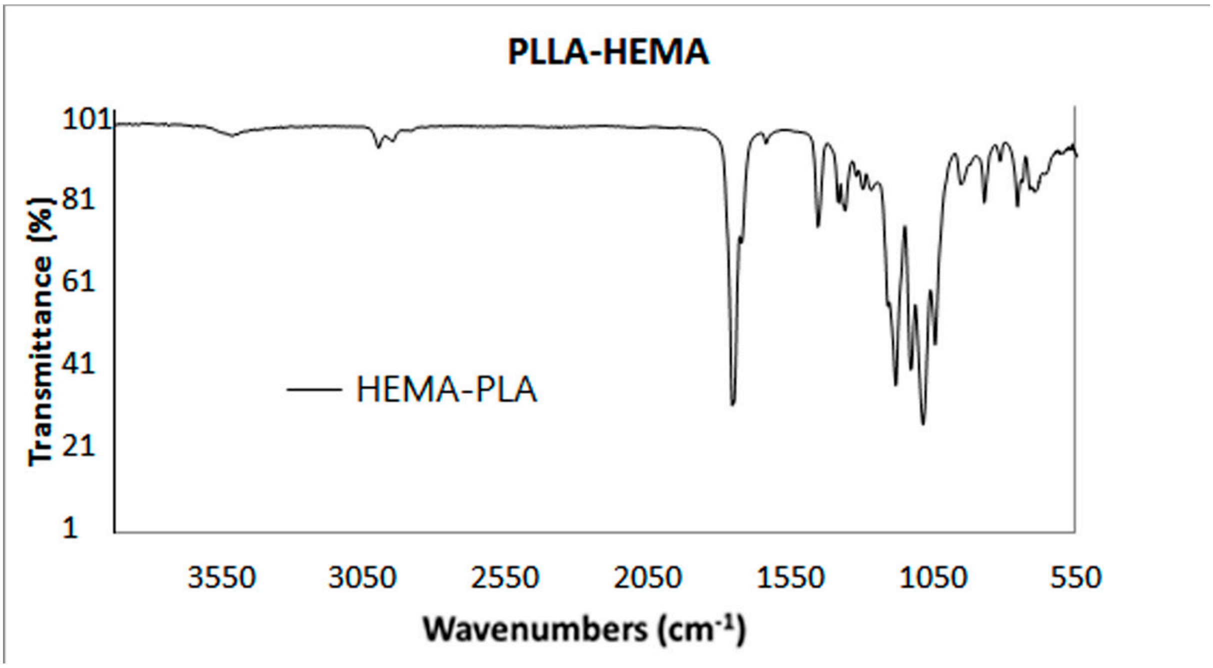

Figure S2. FTIR spectrum of HEMA-PLA macromonomer. The FT-IR spectrum shows significant functional groups: HO (PLA) at 3000-3500 cm^{-1}, C-H (MA and LA) at 2997.54 cm^{-1}, C=O (MA and LA) at 1779.03 cm^{-1}, C=C bond (MA) at 1648 cm^{-1}, and -C-O-C (LA) at 1095 cm^{-1}.

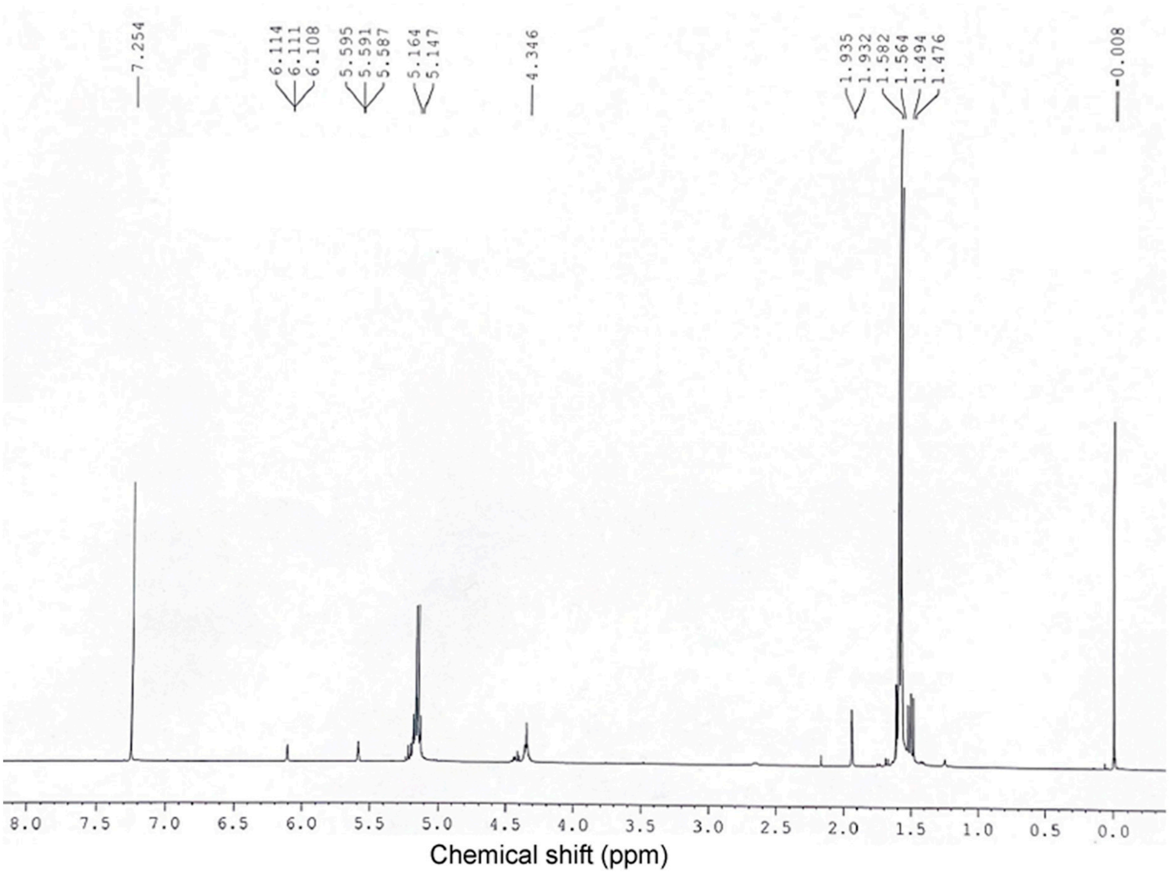

Figure S3. Proton NMR spectrum of HEMA-PLA. ^{1}H NMR (400 MHz, $CDCl_3$, δ, ppm): 1.56-1.58 (d, CH_3, lactide), 1.93 (s, 3H, methacrylate), 4.348 (m, 1H, CH, terminal lactide), 4.362 (m, 4H, CH_2CH_2, methacrylate), 5.130 - 5.182 (m, 1H, lactide), 5.59 (s, 1H, methacrylate), 6.111 (s, 1H, methacrylate).

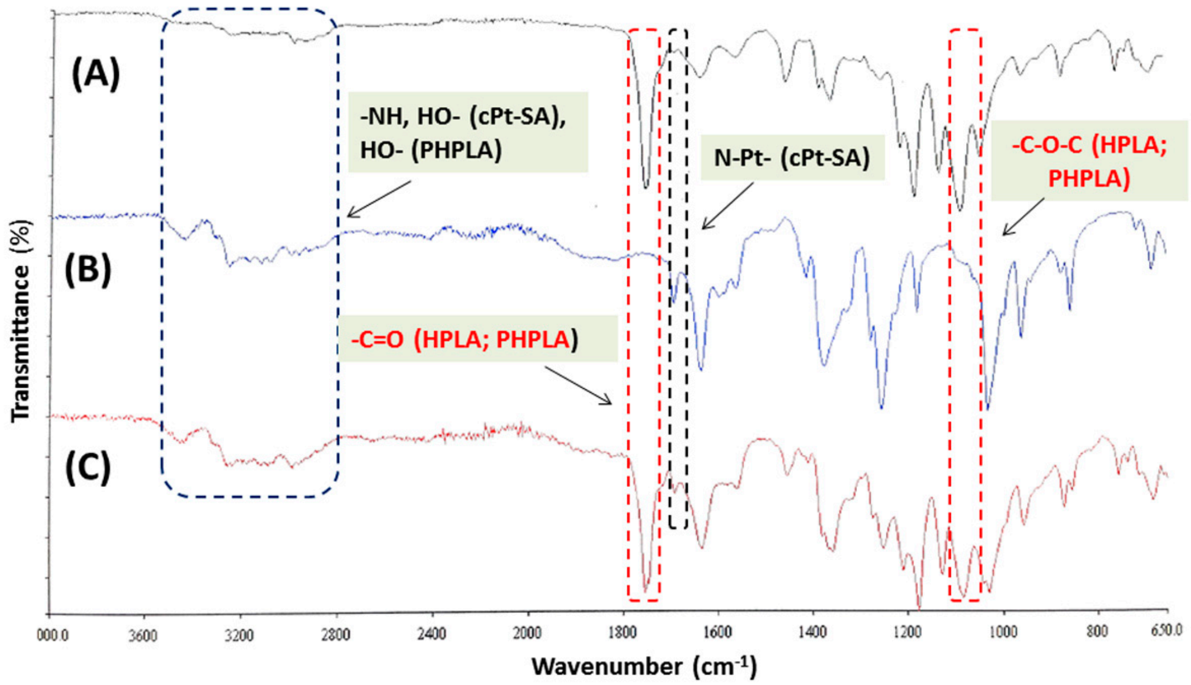

Figure S4. FTIR spectra of PHPLA-cPt copolymer (A), SA-cPt conjugate (B), and HPLA-cPt conjugate (C). FTIR was obtained through the use of powder samples.

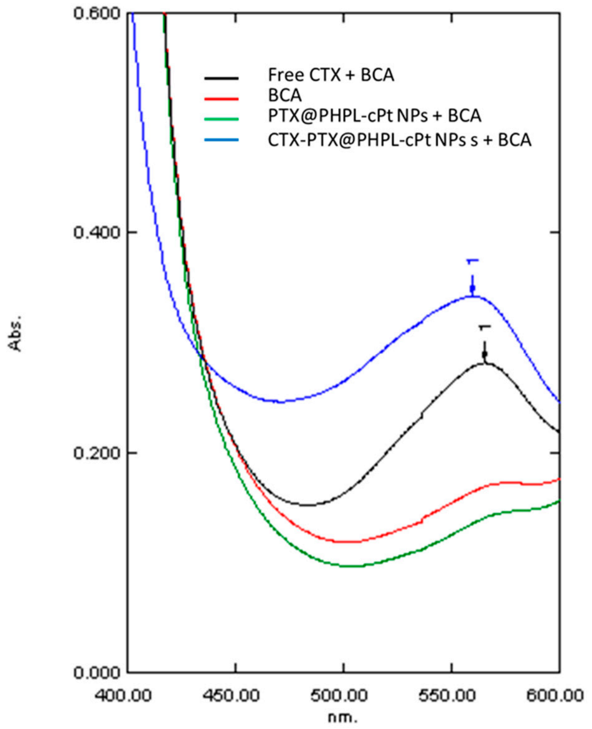

Figure S5. Confirmation of attachment of antibody (CTX) to the nanoparticles using BCA reagent by UV-spectrophotometer**.**
